# Chaperone-Mediated Autophagy and Its Emerging Role in Hematological Malignancies

**DOI:** 10.3390/cells8101260

**Published:** 2019-10-16

**Authors:** Guillaume Robert, Arnaud Jacquel, Patrick Auberger

**Affiliations:** Mediterranean Center for Molecular Medicine, Université Nice Côte d’Azur, C3M/Inserm1065, 06100 Nice, France

**Keywords:** chaperone mediated autophagy, hematological malignancies, lysosome, protein degradation, CMA targeting molecules

## Abstract

Chaperone-mediated autophagy (CMA) ensures the selective degradation of cellular proteins endowed with a KFERQ-like motif by lysosomes. It is estimated that 30% of all cellular proteins can be directed to the lysosome for CMA degradation, but only a few substrates have been formally identified so far. Mechanistically, the KFERQ-like motifs present in substrate proteins are recognized by the molecular chaperone Hsc70c (Heat shock cognate 71 kDa protein cytosolic), also known as HSPA8, and directed to LAMP2A, which acts as the CMA receptor at the lysosomal surface. Following linearization, the protein substrate is next transported to the lumen of the lysosomes, where it is degraded by resident proteases, mainly cathepsins and eventually recycled to sustain cellular homeostasis. CMA is induced by different stress conditions, including energy deprivation that also activates macro-autophagy (MA), that may make it difficult to decipher the relative impact of both pathways on cellular homeostasis. Besides common inducing triggers, CMA and MA might be induced as compensatory mechanisms when either mechanism is altered, as it is the often the case in different pathological settings. Therefore, CMA activation can compensate for alterations of MA and vice versa. In this context, these compensatory mechanisms, when occurring, may be targeted for therapeutic purposes. Both processes have received particular attention from scientists and clinicians, since modulation of MA and CMA may have a profound impact on cellular proteostasis, metabolism, death, differentiation, and survival and, as such, could be targeted for therapeutic intervention in degenerative and immune diseases, as well as in cancer, including hematopoietic malignancies. The role of MA in cancer initiation and progression is now well established, but whether and how CMA is involved in tumorigenesis has been only sparsely explored. In the present review, we encompass the description of the mechanisms involved in CMA, its function in the physiology and pathogenesis of hematopoietic cells, its emerging role in cancer initiation and development, and, finally, the potential therapeutic opportunity to target CMA or CMA-mediated compensatory mechanisms in hematological malignancies.

## 1. General Introduction

Cellular protein homeostasis, also called proteostasis, ensures the integrity of the proteome in multicellular organisms [1]. The maintenance of protein homeostasis is orchestrated by a complex, integrated and interconnected network of mechanisms that control protein fate all along their life into the cell, from their initial synthesis to their final degradation [2]. Proteostasis is closely connected to the signaling pathways involved in the detection of all forms of cellular stress that are capable of altering the proteome integrity [1]. This network comprises all the processes involved in RNA and protein synthesis and catabolism, including gene transcription, RNA translation and metabolism, protein synthesis, and acquisition of the ternary structure of proteins, as well as all their post-translational regulations. It also includes the entire molecular web that regulates protein interactions, the mechanisms associated with the transport and localization of proteins in a defined subcellular compartment, and all the cellular processes implicated in protein catabolism. 

In all organisms, cellular proteins are constantly synthetized and degraded by the cell to maintain cellular homeostasis [3]. The processes of degradation and quality control of proteins are therefore crucial for determining cellular destiny. However, efficiency of proteostasis gradually declines with age, leading to increased cellular alterations, acceleration of cell ageing, neurodegenerative diseases, immune disorders, and initiation and completion of tumorigenesis. 

In subsets of hematological malignancies, whose incidence is intimately associated with ageing, it seems, therefore, essential to investigate (i) the potential defects of protein degradation, (ii) how alteration in proteostasis participates to leukemogenesis, and (iii) whether the implementation of compensatory processes induced by alteration of proteostasis can be targeted for therapeutic purposes. 

As the increasing role of macroautophagy (MA) in leukemogenesis has been recently treated [4], the present review will more specifically encompass the description of the mechanisms involved in CMA, the role of this highly specific degradative process in the physiology and pathogenesis of hematopoietic cells, its emerging function in cancer initiation and development, and, finally, the potential therapeutic opportunity to target CMA or CMA-mediated compensatory mechanisms in cancer and, more specially, in hematopoietic malignancies.

## 2. Protein Renewal and Deciphering of the Main Cellular Catabolic Pathways

**Figure 1:**
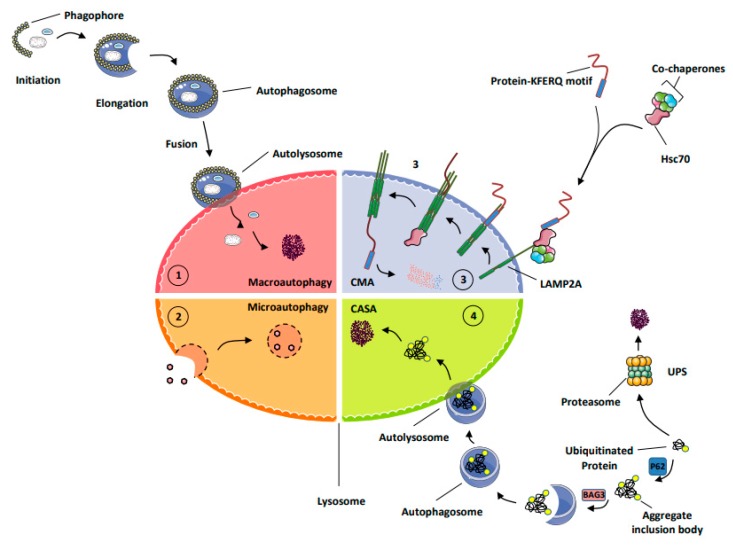
Schematic representation of the main processes of protein degradation. (**1**) MA (macro-autophagy) triggers the degradation of proteins, protein aggregates, lipids, and carbohydrates but also damaged organelles, as well as intracellular micro-organisms into the lysosomes. (**2**) Micro-autophagy corresponds to a less-selective form of autophagy that is carried out through the invagination of the lysosomal membrane around the material to be degraded. (**3**) Chaperone-mediated autophagy (CMA) allows the degradation of cytosolic proteins endowed with a KFERQ-like motif. During CMA, this motif is recognized by Hsc70, also called HSPA8, which triggers their unfolding and subsequent transport into the lysosome, where they are ultimately processed by lysosomal proteases. (**4**) Chaperone-assisted selective autophagy (CASA) ensures the selective ubiquitin-dependent degradation of dysfunctional chaperone-bound proteins in lysosomes. The ubiquitin proteasome system (UPS) is the cellular process by which short-lived proteins and dysfunctional or unfolded proteins are addressed to the proteasome for degradation.

The description of lysosomes by Christian De Duve in 1955 marked a major breakthrough in the understanding of protein catabolism and was the cornerstone of the subsequent discovery of autophagy [5]. Forty years later, a second pathway of protein degradation, the ubiquitin-protein system (UPS), responsible for the degradation of short-lived proteins, was identified [6]. The term micro-autophagy was introduced by de Duve and Wattiaux at the end of the sixties to describe a process involving the invagination of the lysosomal membrane to sequestrate small cytoplasmic constituents for degradation [7]. Following these initial observations, transmission electron microscopy allowed for the identification of membrane structures and multi-membrane vesicles, now called phagophores and autophagosomes and known to be essential elements for the initiation, nucleation, and completion of macro-autophagy (MA). Chaperone-mediated autophagy (CMA) was described more recently and referred to a selective mechanism of degradation of protein substrates bearing a KFERQ-like motif in their amino-acid sequences [8]. To date, the main mechanisms involved in protein catabolism and the regulation of protein homeostasis include the following (Figure 1).

1. MA (macro-autophagy): this process allows the degradation of long-lived proteins, protein aggregates, lipids, and carbohydrates but also damaged organelles, as well as intracellular micro-organisms into the lysosomes. During autophagy, the material to be degraded is engulfed in double-membrane vesicles, called autophagosomes, that fuse with lysosomes and are degraded by a large set of hydrolases and potentially recycled to sustain cell survival [9].
2. Micro-autophagy corresponds to a less-selective form of autophagy that takes place via the invagination of the lysosomal membrane around the material to be degraded. This form of autophagy concerns a minority of proteins and remains sparsely described [10,11].
3. Chaperone-mediated autophagy (CMA) represents a highly selective process of degradation of cytosolic proteins endowed with a KFERQ or KFERQ-like motif in their amino-acid sequences. During CMA, the KFERQ motif present in protein substrates is recognized by the cytosolic chaperone heat-shock protein cognate protein Hsc70c, also called HSPA8, responsible for their unfolding and subsequent transport to LAMP2A (lysosomal-associated membrane protein 2A), which serves as the specific receptor for CMA. Proteins transported to the lysosomal lumen are ultimately degraded by lysosomal proteases, and the products of degradation (amino acids) are potentially recycled to maintain cellular homeostasis and/or promote survival [9].
4. Chaperone-assisted selective autophagy (CASA) ensures cellular protein quality control and, as such, allows the selective ubiquitin-dependent degradation of dysfunctional chaperone-bound proteins in lysosomes. The ubiquitinated proteins are next engulfed in autophagosomes and delivered to lysosomes for degradation [12].
5. The ubiquitin proteasome system (UPS) is the mechanism by which short-lived proteins and dysfunctional or unfolded proteins are addressed to the proteasome for degradation and potential recycling [13].

## 3. Chaperone-Mediated Autophagy (CMA)

### 3.1. Introduction

CMA represents one of the major pathways for protein degradation. This mode of autophagy is involved in the selective degradation of soluble cytosolic proteins. It is estimated that one third of all cytoplasmic proteins are subjected to this specific pathway of degradation. CMA is the only catabolic process in which protein targets are directly translocated through the membrane to the lumen of lysosomes to be ultimately degraded by lysosomal proteases at acidic pH [3].

### 3.2. Mechanisms of CMA

Under adverse conditions such as hypoxia, prolonged starvation [14], oxidative stress [15], or DNA damage, the chaperone Hsc70 binds different co-chaperones, such as Hsp40 (heat-shock protein of 40 kDa), Hip (Hsp70-interacting protein), Hop (Hsp70/Hsp90-organizing protein), Cdc48 (Cell division cycle 48), Bag1 (Bcl2-associated athanogene 1), and Chip (Carboxyl terminus of Hsc70-interacting protein) to form a complex promoting the activity and the ability of Hsc70 to link a KFERQ-like motif in substrate proteins [16,17]. Other chaperones, including Hsp90 (heat-shock protein of 90 kDa), can bind at the same time to this complex, and also bind to an unfolded region of substrate proteins to prevent it from aggregation during cycles of binding/release from Hsc70. Thanks to its ATPase activity, Hsc70 hydrolyzes one molecule of ATP and adopts an ADP-bound state, in order to bind the KFERQ-like motif of target proteins. The macromolecular complex constituted by Hsc70, its co-chaperones, and the protein substrate are next routed to the lysosome, where the direct interaction between Hsc70 and the cytosolic domain of LAMP2A docks the complex to the lysosome. Following the binding of the complex, the protein substrate interacts with the cytosolic domain of LAMP2A. When the protein substrate binds the lysosome, LAMP2A is in a monomeric state and located into lipid rafts, which correspond to a rigid organization of the lysosomal membrane. CMA induction triggered by this binding excludes LAMP2A from the lipid rafts and redirects it to a more fluid membrane area, where LAMP2A first dimerizes and next trimerizes to form a channel that allows the transport of the protein substrate into the lumen of the lysosome. 

**Figure 2:**
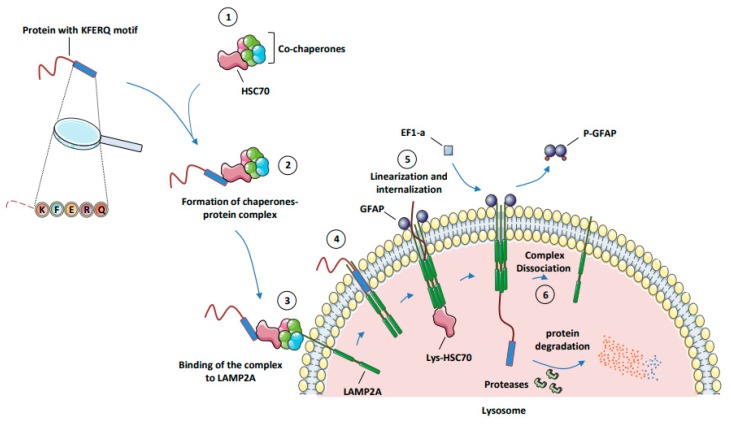
The different steps of CMA. (**1**) Formation of protein complex (Hsc70 + co-chaperones). (**2**) Formation of the chaperone protein complex (Hsc70 complex + protein substrate). (**3**) Binding of the substrate–chaperone complex to LAMP2A. (**4**) Assembly of LAMP2A subunits to form a channel in the lysosomal membrane. (**5**) Linearization, internalization, and degradation of the protein substrate into the lysosome. (**6**) Dissociation of LAMP2A multimer. Abbreviations: CMA, chaperone-mediated autophagy; EF1-α, elongation factor 1 α; GFAP, glial fibrillary acidic protein; Hsc70, heat-shock cognate protein of 70 kDa; LAMP2A, lysosome-associated membrane protein type 2A; Lys-Hsc70, lysosome-associated hsc70.

This complex is stabilized, on the one hand, by the lysosomal fraction of Hsp90 and, on other hand, by GFAP (glial fibrillary acidic protein) factors. GFAP factors are required for the opening and stabilization of the channel formed by the active multiprotein complex. At this step, the protein substrate is linearized and translocated through the channel formed by the LAMP2A homotrimer in the lysosomal membrane [18,19]. A significant part of the Hsc70 pool is present in the lumen of the lysosome and called lysosomal-Hsc70 (Lys-Hsc70). The Lys-Hsc70 contributes to the translocation of the protein substrate from its entry into the channel until its distribution into the lumen of the lysosome. Once present in the lumen of the lysosome, the protein substrate is readily degraded by lysosomal proteases, and the degradation products can be exported to the cytoplasm by lysosomal permeases [9]. At the end of the translocation process from the cytoplasm to the lumen of the lysosome, the elongation factor EF1-α (Elongation Factor 1-α) binds and phosphorylates GFAP. The phosphorylated form of GFAP exhibited a poor affinity for LAMP2A, which favors GFAP/LAMP2A protein complex dissociation and the concomitant formation of p-GFAP dimers. The direct consequence of the loss of GFAP protein at the external membrane of the lysosome is the dissociation of the channel formed by the hetero-trimer of LAMP2A. At this step, monomers of LAMP2A are subjected to degradation or recycled in micro-vesicles that provide a permanent pool of protein that can be rapidly mobilized within the lysosomal surface for future CMA induction (Figure 2).

### 3.3. Modulation of CMA

As MA, CMA is modulated by different forms of cellular stresses, including energy deprivation and oxidative stress, but besides these well-documented effects of different forms of cellular stresses on CMA, new mechanisms of regulation have recently emerged that might be more specific for CMA than for other autophagy processes. The rate-limiting step of CMA is the abundance and assembly of LAMP2A at the lysosomal membrane. Recent studies indicate that LAMP2A stability is controlled by the presence of DJ-1/PARK7 (Parkinsonism-associated deglycase), since DJ-1 deficiency is associated with a higher degradation rate of LAMP2A in Parkinson disease (PD) [20].

The Calcineurin–NFAT pathway was initially identified as the first mechanism able to induce CMA activation in T cells. More recently, this pathway was also identified as one of the mechanism of CMA activation under oxidative stress [15,21]. Indeed, the ROS produced during T-cell activation triggers the nuclear translocation of NFAT1 that binds directly to the lamp2a proximal promoter region, leading to an increase of LAMP2a mRNA expression and CMA activation. Massey et al. described that CMA can be activated under low oxidative stress conditions. In addition, they also reported that LAMP2A^-/-^ cells are statistically more sensitive to oxidative stress, such as H2O2, Paraquat, or Cadmium, compared to LAMP2A^+/+^ cells [22]. Note that these cells are not more sensitive to other modes of stress, such as heat shock or serum removal. These data highlight that CMA plays a crucial role in the adaptive response to the ROS and that other processes; for instance, MA can compensate for nutriment deprivation or changes in external physical parameters. Finally, it was recently reported that, under oxydative stress conditions, LAMP2a expression can be transcriptionally regulated by the transcription factor NFE2L2/NRF2 [23].

Recent results from Maria’s Cuervo group have also highlighted the important role of RARα (retinoic acid receptor alpha) in the regulation of LAMP2A expression and CMA. The RARα activator, ATRA (all-trans retinoic acid), which is used as the leading treatment for patients suffering from acute promyelocytic leukemia (APL), a hematopoietic malignancy characterized by the t(15;17) translocation that juxtaposes PML (promyelocytic leukemia) to RARα, which is a potent inhibitor of LAMP2A expression and, accordingly, of CMA.

Another signaling pathway capable of inhibiting CMA is the mTORC2/Akt (mammalian Target of Rapamycin Complex 2/Protein Kinase B) pathway. mTOR is a serine/threonine kinase activated in a wide range of settings, including alteration in energy levels, amino-acid deprivation, or different forms of cellular stresses. It was recently reported that the lysosomal mTORC2/Akt pathway dampens the assembly of LAMP2A at the lysosomal membrane and therefore inhibits CMA [24]. This inhibitory effect is mediated by the phosphorylation of GFAP by Akt. In energy-deprivation conditions, the PHLPP1 phosphatase (PH domain and leucine-rich repeat protein phosphatase 1) is recruited to the lysosomes to inhibit Akt activation, increasing LAMP2A stability and, consequently, CMA activity [24]. 

Another condition that may impact CMA is endoplasmic reticulum (ER) stress. Although it has long been recognized that ER stress affects MA, the knowledge of the effect that ER stress exerts on CMA is just emerging. Indeed, Li et al., highlighted a new signaling pathway by which ER stress triggers p38 mitogen-activated protein kinase (p38MAPK, MK14) activation and CMA. They demonstrate that triggering ER stress by different means induces a PERK-dependent (PKR-like ER protein kinase) recruitment of mitogen-activated protein kinase kinase 4 (MKK4) and the consecutive activation of a p38MAPK pool at the lysosomal membrane [25]. P38MAPK activation leads to the phosphorylation of LAMP2A at Thr211 and Thr213. This phosphorylation increases both the stability of LAMP2A and its oligomerization at the lysosomal membrane. This new mechanism, called ERICA for “ER-stress-induced CMA” reveals an exquisite relationship between ER-stress and CMA. Note that ERICA seems to be specific to CMA over MA and may offer new therapeutic opportunities to selectively target CMA without impacting MA in different cell types, tissues, and diseases.

### 3.4. CMA Substrates

It is estimated that about 30% of all cellular proteins carry the “so called” KFERQ motif necessary for degradation through CMA which may represent dozens of thousands candidate proteins, but only a very minor fraction of these substrates have been identified so far. Indeed, only two dozen of “true” CMA substrates have been listed, and they are depicted in Table 1 [26]. Among them, AF1Q (ALL1 fused gene from chromosome 1q) [27], PED (phosphoprotein enriched in diabetes) [28], NCor (nuclear receptor co-repressor 1) [29], Vav1 (vav guanine nucleotide exchange factor 1) [30], PKM2 (pyruvate kinase M2) [31], HK2 (hexokinase 2) [32], G6PD (glucose-6 phosphate dehydrogenase) [33], GAPDH (glyceraldehyde-3-phosphate dehydrogenase) [34], Eps8 (epidermal growth factor receptor kinase 8 substrate) [35], Rnd3 (Rho family GTPase 3) [36], mutant P53 [37], SNCA (α-synuclein) [38], HIF1α (hypoxia-inducible factor alpha) [39], MEF2A and MEF2D (myocyte-enhancer factor 2A and 2D) [40,41], MAPT (microtubule associated protein tau) [42], HTT (huntingtin) [43], c-Myc (myc proto-oncogene) [44], TFEB (transcription factor with an EB-Box) [45], PUMA (P53 upregulated modulator of apoptosis) [46], and Bcl2-L10 (Bcl2-like10) [47] were formally identified or at least proposed as CMA substrates. Finally, other CMA substrates are also mentioned in Table 1.

Several CMA substrates identified so far, including PKM2 (pyruvate kinase M2), GAPDH (glyceraldehyde-3 phosphate dehydrogenase), HK2 (Hexokinase 2), and G6PD (glucose-6 phosphate dehydrogenase) are metabolic enzymes, and degradation of these enzymes through CMA could contribute to the inhibition of metabolic activity and glycolysis. G6PD catalyzes the first and limiting step of the pentose pathway, generating 6-phosphoglucono-d-lactone from G6P. HK2 phosphorylates glucose to produce glucose-6-phosphate. PKM catalyzes the last and limiting step of glycolysis. GAPDH catalyzes the sixth step of glycolysis to produce 1,3-bisphosphoglycerate from glyceraldehyde-3-phosphate. Other CMA substrates, such as synuclein α, tau, and huntingtin, are involved in different brain cellular functions, and the alteration of the expression of these factors is linked to diverse degenerative diseases, including Parkinson disease (PD), Alzheimer’s disease (AD), and Huntington disease (HD).

Very recently, a quantitative multiplex analysis of CMA substrates performed in a wide range of cancer cell lines identified more than 250 putative new CMA substrates [48]. This was performed in a condition where CMA was inhibited through the expression of a highly specific LAMP2A siRNA in order to specifically inhibit CMA without directly affecting MA. Among the newly discovered substrates, a dozen eukaryotic initiation translation factors including, EIF2A, EIF2D, EIF3B, EIF4A1, EIF4G1, EIF4G2, EIF4G3, EIF4H, and EIF5, were significantly enriched in the lysosomal fraction, linking CMA inhibition to increased translational regulation. In addition, they established that components of the cellular translation machinery were bona fide CMA substrates in multiple cancer cell lines, and the degradation of several of these factors by CMA was confirmed by functional assays. Note that most of the identified CMA substrates display high expression in multiple primary cancers compared to their normal healthy-tissues counterparts. Finally, CMA activation leads to degradation of most of these substrates associated with a concomitant inhibition of translation.

### 3.5. Crosstalk Regulation between MA and CMA

MA and CMA are two subtypes of autophagy that play a critical role in cellular quality control, under physiological and pathological conditions [49,50,51,52,53]. It was reported that, when MA was blocked in cells, CMA activity was induced, which leads to cell survival. It is well established that defects in CMA can also be compensated for by increased MA in adverse conditions [22,50,51,52,53]. The mechanism involved in this co-regulation was recently elucidated. Indeed, Wang et al. reported that Unc-51 like autophagy activating kinase1 (ULK1) could be phosphorylated by PKCα in nutriment-rich condition, enhancing its affinity for Hsc70 and increasing its degradation by CMA. This degradation of ULK1, a key component of MA suppresses autophagy process [54]. In agreement with this concept, it was also demonstrated that pharmacological inhibitors of PKCs induce autophagy [55]. In conclusion, MA and CMA are two finely regulated processes of protein degradation which can complement each other.

### 3.6. Crosstalk between UPS and CMA

As described above, it is well established that CMA activation can compensate for alterations of MA and vice versa. In the same line, a significant interaction between CMA and the UPS does exist. Maria Cuervo’s team convincingly demonstrated through the use of a CMA fluorescent reporter that pharmacological inhibition of the proteasome by MG132 led to an upregulation of CMA in murine fibroblast cells [56]. It is noteworthy that inhibition of the proteasome pathway by chemical compounds leads to an increase of CMA but that invalidation of CMA triggers a decrease in proteasomal activity. This decrease in proteosomal activity is likely due to alteration in the turnover of some proteasomal subunits leading to a defect in proteasomal assembly. 

## 4. Physiological and Pathological Functions of CMA

The protein level of LAMP2 and, by consequence, of LAMP2A decreases with age and correlates with a reduction of MA and CMA that could reflect the greater incidence of neurogenerative disorders and cancer in elderly patients. It is well established that the levels of LAMP2 mRNA and protein gradually decline in peripheral blood cells until 60 years. Importantly, incidence of myelodysplastic syndrome and acute myeloid leukemia increases in the general population with ageing [57,58,59]. Whether or not increased incidence of MDS/AML with age is related to reduction of LAMP2 expression has not been explored. Nevertheless, several lines of evidence indicate that loss of MA and CMA that occurs during ageing reduces the ability of neuronal cells to efficiently eliminate protein aggregates, a condition that may favor the onset of neurodegenerative disorders, including PD, AD, and HD [60,61,62,63,64,65]. In the same line, defects of oncogenic protein elimination, normally managed through MA or CMA, may promote tumorigenesis and hematological malignancies.

The global invalidation of LAMP2 in mice is responsible for a high level of mortality. Indeed, 50% of the LAMP-2 knockout male or female mice die before the 40th postnatal day. Furthermore, the surviving mice are smaller than control ones [66]. These mice exhibit strong accumulation of autophagic vacuoles in most of their tissues, including liver, kidney, and pancreas, as well as the cardiac and skeletal muscles. They also present hippocampal dysfunction leading to motor deficits and impaired learning. The set of clinical observations detected in these mice recapitulates most of the symptoms found in human Danon disease, a setting associated with familial mutations in LAMP2 protein. The impact of LAMP2 invalidation has not been well characterized in the hematopoietic compartment, but a huge accumulation of double membrane vacuoles in neutrophils coming from LAMP2^-/-^ mice has been reported by the same authors. Alteration of neutrophils in LAMP2^-/-^ mice favors the development of severe periodontitis early in life. This inflammatory disease is a consequence of a major defect in the function of neutrophils, which is involved in the local defense against bacteria. Finally, one team developed a LAMP2- specific invalidation under the control of the Albumin-CRE promoter in order to study properties of CMA-deficient liver [33]. Authors revealed that these mice exhibit a hepatosteatosis and alteration of glucose metabolism. To the best of our knowledge, no specific invalidation of LAMP2 has been carried out in the hematopoietic compartment.

### 4.1. Function of CMA in Cancer Initiation and Progression

Conversely to the well-admitted role of MA in cancer initiation, progression, and metastasis [4], knowledge of the potential involvement of CMA in tumorigenesis is just emerging. Pioneer studies in the field come once again from Maria’s Cuervo laboratory. In a recent manuscript, Kwon et al. highlighted the role of CMA in the proliferation and survival of cancer cells. They first established a high basal activity of CMA in a wide variety of tumor cells lines and human tumor patient samples without significant modulation of MA. Increased CMA activity was associated with the elevation of LAMP2A lysosomal receptor expression. 

How CMA may impact cancer initiation and progression is poorly understood. However, it is clear that CMA is required for optimal cell growth and tumorigenesis. This drastic effect of CMA on tumorigenesis can be at least in part explained by the profound effect that CMA exerts on cellular metabolism. Inhibition of CMA diminished the glucose-dependent extracellular acidification and reduced glycolysis. However, in CMA-deficient tumor cell lines, oxygen consumption and oxidative metabolism seem to not be affected. Increased aerobic glycolysis, which corresponds to the “Warburg effect”, is one of the hallmarks of tumor cells. The increased glycolysis observed following the inhibition of CMA is responsible for the reduction of energy through inhibition of adenosine triphosphate production and biosynthesis of macromolecules, which are essential for cancer cell proliferation and survival. Taking into account the role of metabolism alteration in cancer initiation and progression, it is not surprising that several well-characterized CMA substrates are indeed metabolic enzymes. Analysis of the TCGA AML cohort (TCGA, NEJM, 2012) indicated that patients exhibiting high PKM and G6PD levels exhibit a bad prognosis and lower overall survival, confirming that overexpression of these metabolic enzymes is linked to tumor growth and leukemia expansion. Whether increase of these metabolic enzyme expressions through inhibition of CMA is involved in tumor initiation and progression will warrant further studies. 

### 4.2. Implication of CMA in Hematological Malignancies

As a quick adaptive degradation process to respond to adverse stress conditions, it is not surprising that alterations of CMA are observed in a variety of human pathologies, but also in normal ageing processes. This decrease in CMA activity was observed in many human and murine cell types during the ageing process [67], a condition associated with increased risk to develop cancer, as exemplified in Myelodysplastic Syndrome (MDS), where age-associated mutations in different genes favor MDS initiation but also transformation into acute myeloid leukemia (AML) [68]. It is well-documented that the decrease of CMA activity during ageing is related to an excessive degradation of the LAMP2 protein. Furthermore, changes in the lipid composition of the lysosomal membrane that appears with age increase the degradation of LAMP2A in the lysosomal lumen [18,69,70]. Therefore, the binding and the translocation of substrate proteins by LAMP2A and CMA activity are clearly reduced in lysosomes of older organisms [45].

In addition, age-related decrease in LAMP2 protein expression may have a profound impact in the hematopoietic compartment. Indeed, LAMP2 transcript and protein levels significantly decreased in peripheral leukocytes of 65–69 (P < 0.01) and over 70 years (P < 0.001) groups compared to 40–44 years group [57]. This observation can be again correlated with the median age of onset of leukemia observed in MDS and AML patients. 

### 4.3. CMA Substrates with a Special Relevance to Hematopoietic Malignancies

#### 4.3.1. AF1Q/MLLT11

AF1Q exhibits a KFERQ-like motif, which is recognized by Hsc70c for CMA-dependent proteolysis. AF1Q, or MLLT11 (myeloid/lymphoid or mixed-lineage leukemia translocated to 1q), is a mixed-lineage leukemia gene fusion partner, identified as a poor prognostic biomarker in pediatric acute myeloid leukemia (AML) and myelodysplastic syndrome (MDS). AF1Q is highly expressed in CD34+ hematopoietic progenitor cells, but its function has not been clearly defined. Pharmacological inhibitors of lysosomal degradation, such as chloroquine, increase AF1Q levels, whereas activators of CMA, including 6-aminonicotinamide and nutrient starvation, decrease AF1Q expression [27]. AF1Q interacts with Hsc70c and LAMP2A, which are core components of the CMA machinery. Knockdown of Hsc70 or LAMP2A increase AF1Q protein levels, whereas overexpression of both proteins produces the opposite effect. 

#### 4.3.2. Bcl2-L10 (Bcl2-Like Protein 10/Bcl-2L10)

Bcl2-L10 also known as Bcl-B, is an anti-apoptotic member of the Bcl2 family, whose physiological function has long remained a conundrum. Recently, Hamouda et al., generated transgenic mice in which Bcl2-L10 expression is driven by the E-mu promoter, specifically in the B cell lineage [71]. These mice develope, around two years of age, a hematological malignancy that accurately recapitulates most of the characteristics of human multiple myeloma (MM). Moreover, splenic B but not T cells from younger transgenic mice (three months) are efficiently protected from spontaneous apoptosis and exhibit a higher efficiency to differentiate into plasmocytes ex vivo, further suggesting an important role of BcL2-L10 in B cell differentiation and plasma cell homeostasis. Regarding myeloid malignancies, we recently established that, in a cohort of one hundred MDS/AML patients, high Bcl2-L10 expression correlated with resistance to azacytidine and a pejorative prognosis [72,73]. Interestingly, Bcl2-L10 bears a putative KFERQ-like (RLKEQ) motif in its amino-acid sequence, suggesting that Bcl2-L10 stability, in addition to its well-established regulation by the ubiquitin proteasome system [74,75], could be modulated by CMA. Several recent reports in the literature also indicate that Bcl2-L10 is involved in the regulation of autophagy by directly interacting with Beclin-1 and through the regulation of mitophagy [76,77,78]. Globally, it is likely that Bcl2-L10 may modulate different forms of autophagy (MA, CMA, Mitophagy) and/or may be modulated by catabolic processes such as CMA or the UPS. However, the identification of Bcl2-L10 as a true CMA substrate requires further experimental confirmation.

#### 4.3.3. c-Myc

c-Myc is an important transcription factor of the helix loop helix family whose expression is persistently increased in many solid and hematopoietic cancers. Myc is frequently amplified in hematological malignancies. Thirty percent of multiple myeloma patients exhibit an amplification of the Myc gene through different fusion mechanisms. Amplification of Myc is also detected in a subset of non-hodgkin lymphoma, including Burkitt lymphoma. In a recent study, Gomes et al. demonstrate that CMA inhibition in fibroblasts increases Myc-driven cellular transformation [44]. Indeed, CMA blockade leads to accumulation of both total and nuclear Myc, promoting cell proliferation and colony formation. Impaired CMA functionality also improves tumorigenesis-related metabolic changes observed upon Myc-transformation. Although not directly degraded through CMA, CMA indirectly impacts cellular Myc levels by controlling its proteasomal degradation. Altogether, these data demonstrate that CMA mitigates Myc oncogenic activity by promoting its proteasomal degradation and reveals a novel tumor-suppressive role for CMA in nontumorigenic cells. 

#### 4.3.4. TP53

TP53 acts as a checkpoint control for cell cycle, cell differentiation, programmed cell death, and DNA synthesis and repair. TP53 is a major tumor suppressor, and its inactivation promotes tumor survival, proliferation, genomic instability, angiogenesis, and metastasis in many human cancers. Most of TP53 inactivation mechanisms involve single-base substitution or loss of alleles. It was previously reported that wild-type TP53 exhibits a KFERQ-like motif and can be degraded by CMA [26]. Importantly, it was established that the degradation of the mutated form of TP53 is specifically mediated by the CMA pathway [37]. This observation offers a unique opportunity to impact the degradation of a powerful tumor suppressor found in many cancers through CMA modulation.

#### 4.3.5. TFEB

TFEB (transcription factor EB) is the master regulator of autophagy and lysosome biogenesis. It binds to a CLEAR (coordinated lysosomal expression and regulation) responsive element in the promoter region of its target genes, upon translocation into the nucleus [79]. Many of the TFEB-regulated genes are involved in lysosome biogenesis and regulation of autophagy. In LAMP2A-deficient mice, the TFEB expression level is elevated likely due to an inhibition of CMA [45], highlighting TFEB as a CMA substrate. Accumulation of TFEB in CMA-deficient cells can explain, at least in part, why blocking CMA increases MA. In agreement with the notion that TFEB behaves as a CMA substrate, several KFERQ-like motifs are present in the TFEB amino-acid sequence. 

#### 4.3.6. IκB

NF-κB is a transcription factor (TF) formed by a heterodimer composed by p50 and p65 proteins. This TF is able to induce the expression of genes mainly involved in immune and inflammatory responses [80]. In normal condition, NF-κB is sequestered into the cytosol by the inhibitory factor IκB [81]. Under stress conditions, IκB is degraded, allowing NF-κB to migrate in the nucleus to exert its role on gene transcription. The mechanisms of IκB degradation involve different processes, such as the UPS, calpains, caspases, or nuclear proteases, depending on the cell type and the initial stimulus. Importantly, in nutriment-deprivation conditions, CMA triggers the specific degradation of IκB, allowing NF-κB activation and its translocation to the nucleus [82].

#### 4.3.7. PKM2

The M2 isoform of pyruvate kinase is generated by alternative splicing of the PK mRNA. The dimeric and tetrameric forms of PKM2 are less active than the corresponding complexes formed by the PKM1 isoform. PKM2 in its dimeric state engages the glycolysis intermediates toward glucose biosynthesis, whereas, in its tetrameric form, PKM2 promotes glycolysis for ATP production. PKM2 is found overexpressed in many cancers and high PKM2 levels correlated with a poor clinical outcome [83,84,85,86]. PKM2 strongly contributes to cancer cell metabolism by reducing oxidative metabolism, thus favoring tumor growth in a hypoxic environment [87]. In a normal or low-glucose environment, inhibition of PKM2 expression level or enzymatic activity by RNA interference or pharmacological inhibitors, such as shikonin [88,89], efficiently reduces cell survival and tumor growth. In a recent study, Wei et al. demonstrated that high glucose concentrations promote acetylation of PKM2 on Lys305 residue, leading to a decrease in PKM2 activity and degradation of PKM2 by CMA [31]. Thus, induction of PKM2 degradation by CMA may represent a promising strategy for targeting and eradicating cancer cells. 

#### 4.3.8. HK2

Hexokinase II (HK2) is a key enzyme that catalyzed the first step of glucose metabolism. HK2 is an oncogenic kinase which strikingly contributes to initiation and maintenance of tumorigenesis [90]. Many oncogenes are known to induce HK2 transcription, including TP53, c-Myc, and HIF-1α. A recent article pointed out the role of CMA in the degradation of HK2 [32]. In addition, two different teams used a similar approach based on drug combination to efficiently block MA as a means of increasing CMA. Indeed, the concomitant blockade of Fms-like tyrosine kinase 3 (FLT3) using Quizartinib (a tyrosine kinase inhibitor) and MA by means of small molecules (C43 or TAK-165) identified in a functional screen triggered HK2 degradation and metabolic catastrophe in solid and hematopoietic cancer cells [32,91]. These combined treatments may represent a pertinent approach to induce metabolic catastrophe and death of tumor cells through CMA-mediated HK2 degradation. In this context, degradation of HK2 through CMA activation appears as a pertinent strategy to inhibit tumor formation. 

#### 4.3.9. Elimination of Fusion Protein by MA

It was also established that some fusion proteins found in hematopoietic malignancies, including p210BCR-ABL and PML-RARα which are drivers for chronic myeloid leukemia and acute promyelocytic leukemia, respectively, can be degraded through MA [92,93,94] or other mechanisms [95,96]. In the same line, recent data established the p210BCR-ABL fusion protein can be degraded through CMA. In this context, it would be of prominent interest to determine whether some of the fusion proteins responsible for hematological malignancies, including PML-RARα, could also be degraded through CMA. If so, increasing CMA using the abovementioned combination of drugs could represent a pertinent therapeutic strategy for cancer treatment and, more particularly, for FLT3-mutated AML.

## 5. Targeting CMA in Hematological Malignancies

### 5.1. Small Molecules Compounds that Affect CMA

It is clearly established that CMA can favor cancer development. For instance, CMA inhibition blocks tumor growth and reduces metastasis formation in human lung cancer xenografts mice model [97]. Furthermore, it was also reported that CMA is upregulated and required for the survival of breast cancer cells [98]. Contrarywise, activation of CMA in hematopoietic malignancies contributes to eliminate oncogenes, such as mutated TP53, AF1Q, PKM2, or fusion proteins such as P210BCR-ABL. We reported recently that low LAMP2 expression levels that contribute to decreased CMA activity were associated with a poor overall survival in AML patients [47]. In this context, developing tools able to modulate CMA activity in a cell-context-dependent manner might represent a pertinent therapeutic strategy in solid and hematopoietic cancers.

We previously emphasized that CMA and MA are mechanistically linked. Indeed, CMA can compensate for alteration of MA and vice versa. In this line, Finn et al. investigated different well-described inhibitors in order to find molecules able to specifically impact CMA without affecting MA [99]. From their results, it seems clear that the conventional MA activator (Rapamycin) or inhibitor (3-methyladenine) had no impact on CMA. However, these authors identified three molecules able to inhibit the CMA process. Among them, two, Cycloheximide and Anisomycin, are known to block protein synthesis, and the third one is the p38 MAPK inhibitor, SB230580. In this screen, two compounds able to induce CMA were also identified. The first one, 6-aminonicotinamide, is a G6PDH inhibitor, and the second one, Geldanamycine, is a selective inhibitor of the chaperone HSP90 (Table 2). To bring the proof of concept that HSP90 inhibition could be a pertinent strategy to induce CMA in hematological malignancies, Allende-Vega et al. established that, under stress conditions induced by Dichloroacetate, a PDK1 inhibitor, 17-Allylamino Geldanamycin (17-AAG), a Geldanamycin derivative used in the clinic, favors the degradation of the mutant form of TP53 (R248Q) through CMA in AML cell lines [100]. Both studies confirmed that HSP90 acts as an inhibitor of the CMA process and highlighted inhibition of this protein as a pertinent strategy to specifically increase CMA.

As mentioned previously, the RARα signaling pathway negatively regulates LAMP2A expression, but also RAB11 and RILP levels two proteins involved in LAMP2A trafficking to lysosomes [101,102]. The use of synthetic derivatives of all-*trans* retinoic acid (ATRA) that specifically block the inhibitory effect of RARα on CMA process without affecting its transcriptional program makes cells more resistant to oxidative stress and proteotoxicity, which could have a real impact, especially in hematopoietic malignancies and, more particularly, in APL [101]. 

Several teams reported a decrease of PHLPP1 in chronic lymphocytic leukemia (CLL) [103,104]. This protein acts as a negative regulator of the TORC2/AKT pathway, which is involved in the inhibition of CMA [24]. In CLL, the use of a TORC2 inhibitor (Torin-1) or an AKT inhibitor restores active CMA and degradation of pro-oncogenic proteins involved in the initiation and development of cancer [24,105]. In addition, two recent studies reported the synergic effect of the combination of FLT3 (AC220/Quizartinib) and autophagy inhibitors (C43/spautin-1) [32] or (TAK165) [91] to efficiently kill AML cells. This co-treatment, which strikingly increases CMA, triggers the degradation of the mutated form of TP53, GAPDH, IκB-α, and HK2 proteins with the final consequence to promote metabolic catastrophe and cell death of AML cells. In conclusion, the strategy aimed at increasing CMA activity appears as an attractive therapeutic approach to treat hematological malignancies either alone or in combination with their conventional chemotherapy.

### 5.2. Potential Role of CMA in APL Cell Differentiation and Treatment

As mentioned above, RARα activation inhibits LAMP2A expression and, as a consequence, CMA. This is likely of utmost importance in APL. Indeed, ATRA activates PML-RARα in APL, leading to inhibition of proliferation and induction of neutrophilic differentiation, and therefore cures most patients suffering from this hematopoietic malignancy. Several lines of evidence indicate that MA is involved in ATRA-mediated differentiation of the APL NB4 cell line [106,107,108,109,110]. If MA definitely plays a role in the differentiation of APL cell lines and bone marrow cells from APL patients, the impact of CMA in this process has not been explored. ATRA has revolutionized the management and treatment of patients suffering from APL, leading to cure in 70% of patients and in nearly 95% of patients when combined with Arsenic Trioxide. Since ATRA was shown to significantly reduce the level of LAMP2A expression and, as a consequence, to dampen CMA, it would be of particular interest to decipher the role of CMA and to analyze the impact of blocking CMA on neutrophilic differentiation of bone marrow cells from APL patients.

## 6. Conclusions and Outlook

In this review, we discussed how CMA is involved in, and regulated during, hematopoietic cell differentiation and how it can impact leukemogenesis. As autophagy is known to play an important role in the process of hematopoietic cell differentiation [4,111,112,113], it was expected that CMA would also be a regulator of this process. However, the role of CMA as an important actor of the proteostatic and metabolic modifications necessary for hematopoietic cell differentiation is just emerging. 

As an energy-consuming process, physiological differentiation of hematopoietic cells requires permanent adaptation of their protein content, which can be achieved by several degradative mechanisms, including CMA. Hematopoietic malignancies are systematically characterized by profound defects in cell differentiation. Restoration of an effective differentiation process in myeloid malignancies has thus emerged as a pertinent therapeutic strategy. This notion is particularly well exemplified by the successful use of ATRA to promote APL differentiation, even leading to cure in a majority of patients. ATRA acts as an inhibitor of CMA through its ability to reduce LAMP2A expression. Therefore, even if the role of CMA in ATRA effects has not been formally studied, it is tempting to speculate that the inhibition of CMA by ATRA may be important for the differentiation of myeloid blasts in APL patients. 

So far, selective modulators of CMA are still lacking, but recent findings in the literature highlighted the possibility to target, more specifically, CMA, using ATRA, for instance. Inhibitors of p38MAPK could also be used in order to dampen CMA, since p38MAPK phosphorylates LAMP2A on two serine residues, leading to an increased accumulation at the lysosomal membrane and activation of CMA. Nevertheless, further investigations should be carried out before small molecule modulators of autophagy can be used for therapeutic purposes in human diseases, including hematological malignancies. Recently, several very promising analogs of chloroquine that efficiently and more specifically inhibit autophagy were developed and have shown impressive effects in some tumor cell lines and xenografted mice models of solid tumors [114,115,116]. These molecules also warrant testing as CMA modulators in hematopoietic malignancies.

## Figures and Tables

**Table 1:** CMA substrates with an established function in cancer.

Symbol	Protein Full Name	Function	Deregulated in:	CMA Substrat Ref:
GAPDH	Glyceraldehyde 3-phosphate deshydrogenase	Carbohydrate Metabolism	Non hodgkin’s B lymphoma	[34]
HK-2	Hexokinase-2	Carbohydrate Metabolism	Ovarian cancer	[32]
PKM2	Pyruvate Kinase M2	Carbohydrate Metabolism	AML, Melanoma	[31]
TP53	Tumor Protein P53	Tumor suppressor protein	Most of cancers	[26]
Mutant TP53	Mutant Tumor Protein P53	Oncogene	Most of cancers	[37]
MDM2	Mouse Double Minute 2 homolog	E3 ubiquitin Ligase	Glioma, ALL, Melanoma	
PUMA	P53 upregulated modulator of apoptosis	BH3-only Pro-Apoptotic protein	Breast, Colon cancers	[46]
AF1Q (MLLT11)	MLLT11 Transcription Factor 7 Cofactor	Oncogene	AML	[27]
c-Myc	MYC Proto-Oncogene, BHLH Transcription Factor	Oncogene	Most of cancers	[44]
IκΒ	NFKB Inhibitor Alpha	NF-κB Inhibitor	B-cell lymphoma	
CHK1	Checkpoint Kinase 1	Cell cycle arrest	Breast, Ovarian Cancers	
Vav1	Vav Guanine Nucleotide Exchange Factor 1	(GEFs) for Rho family GTPases	Pancreatic cancer	[30]
HIF-1α	hypoxia Inducible Factor 1 alpha	Transcriptional regulator of the adaptive response to hypoxia	Lymphoma, colorectal cancers	[39]
NCOR1	Nuclear Receptor Corepressor 1	Promotes histone deacetylation and the formation of repressive chromatin structures	NSCLC, Gastric cancer	[29]
PED	Phosphoprotein Enriched in diabetes	Facilitate glucose transport	Gastric cancer	[28]
EPS8	Epidermal Growth Factor Receptor Pathway Substrate 8	Signaling adaptapter	Pancreatic cancer	[35]
RND3	Rho Family GTPase 3	Negative regulator of cytoskeletal organization	Gastric cancer	[36]
ANXs	Annexins	membrane scaffold, linking Ca2+ signalling to membrane dynamics	Breast Cancer	
TFEB	Transcription Factor EB	Transcription factor of lysosomal genes	Pancreatic, Renal cancers	[45]
EGFR	Epidermal Growth Factor receptor	Receptor tyrosine kinase binding ligands of the EGF family	Head and neck squamous cell carcinoma (HNSCC)	
GAL3	Galectine-3	Numerous cellular function: cell growth, adhesion, mitosis, proliferation and apoptosis	Diffuse large B-cell lymphoma (DLBCL), Prostate, liver cancer	
RKIP	Raf Kinase Inhibitor Protein	Raf Kinase Inhibitor	Prostate cancer	
UBQLN1	Ubiquilin 1	Ubiquitin like protein	Gastric cancer	
Bcl2-L10	Bcl2 Like 10	Anti-apoptotic protein of BCL2 family members	MM, MDS and AML	[47]

**Table 2:** Small molecule modulators of CMA activity.

Compounds	Target	Effect on CMA	Refs
Cycloheximide	Protein synthesis inhibitor	Inhibition	[99]
Anisomycin	Protein synthesis inhibitor	Inhibition	[99]
SB230580	P38 MAPK inhibitor	Inhibition	[99]
Geldanamycin	HSP90 inhibitor	Activation	[99]
17-AAG/DCA	HSP90 inhibitor + PDK1 inhibitor	Activation	[100]
6-aminonicotinamide	G6PDH inhibitor	Activation	[99]
synthetic ATRA derivatives	RAR-alpha inhibitor	Activation	[101]
torin	TORC2 inhibitor	Activation	[24]
TAK165/AC220	MA inhibitor + FLT3 Inhibitor	Activation	[91]
Spautin/AC220	MA inhibitor + FLT3 Inhibitor	Activation	[32]
